# Supplementary material for: Collaborative design of a decision aid for stroke survivors with multimorbidity: a qualitative study in the UK engaging key stakeholders
Source: BMJ Open. 2019 Aug 15;9(8):e030385. doi: 10.1136/bmjopen-2019-030385 (PMC6701575; doi:10.1136/bmjopen-2019-030385)
Supplement: Supplementary data [file bmjopen-2019-030385supp001.pdf]

Box 1: Topic guide for the separate focus groups in the SEM

In a large group, explain:

- Study aim
- What a LHS is, and how a LHS might work in general practice
- The co-production approach we are using

In separate focus groups:

- Ask participants to provide examples of information which are/would be useful for patients/carers/clinicians/health commissioners?
- Previous experiences of information delivery: What worked well? What worked badly/not so well?

Explore stakeholders' understandings around what is data linkage, and what is a learning health system (LHS)?

- How would a LHS work in practice for stroke?
- Any ethical concerns about this process (particularly regarding data linkage)? How can these be addressed?
- What types of information could be generated using this method?
- How broadly might they be delivered?

Discuss ideas for new information interventions

- Feedback from individual groups

Develop as a larger group a priority list for key priorities for data and information needs.

Note: Since a few healthcare professionals could not attend the focus groups, we conducted face-to-face interviews with them using the same topic guide.

Box 2: post-usability interview - patients

- Do you have any comments about today's session? Feel free to comment on anything you want.
- How did it feel having a consultation using the decision aid?
- Who do you think should be involved in making decisions about how acceptable your risks are of having a further stroke?
  - □ your doctor alone
  - □ mostly your doctor
  - □ your doctor and you equally
  - □ mostly you
  - □ you alone
- Who do you think should be involved in making decisions about ways to reduce your risk of stroke?
  - □ your doctor alone
  - □ mostly your doctor
  - □ your doctor and you equally
  - □ mostly you
  - □ you alone
- To what extent do you agree with the following statement? (from 1 strongly disagree to 5 strongly agree)
  - The decision aid will help patients with adopting healthier behaviours, such as changing Lifestyle habits and/or taking medication according to the management plan they agreed on.
  - Having seen how the decision aid works, patients will likely look for more information about stroke and its risk factors.
- (if agreed on statement above) Can you please describe how the decision aid might support patients in changing some of their health-related habits?
- What might make it difficult (barriers, hurdles) for patients to change some of their health-related habits?
- Would you find the decision aid helpful for your own health-related habits?
- What do you <u>like</u> about the decision aid?
- What <u>don't you like</u> about the decision aid?
- What suggestions do you have to improve the decision aid?

Give Acceptability and Usability questionnaires.
